# A 16-Channel Dipole Antenna Array for Human Head Magnetic Resonance Imaging at 10.5 Tesla

**DOI:** 10.3390/s21217250

**Published:** 2021-10-30

**Authors:** Myung Kyun Woo, Lance DelaBarre, Matt Waks, Jerahmie Radder, Uk-Su Choi, Russell Lagore, Kamil Ugurbil, Gregor Adriany

**Affiliations:** 1 Center for Magnetic Resonance Research, University of Minnesota, Minneapolis, MN 55455, USA; dela0087@umn.edu (L.D.); waks0005@umn.edu (M.W.); radd0012@umn.edu (J.R.); rllagore@umn.edu (R.L.); ugurb001@umn.edu (K.U.); adria001@umn.edu (G.A.); 2 Department of Electrical and Computer Engineering, Seoul National University, Gwanak-gu, Seoul 08826, Korea; 3 Gwangju Alzheimer’s Disease and Related Dementia Cohort Research Center, Chosun University, Gwangju 61452, Korea; uschoi@chosun.ac.kr

**Keywords:** antenna coupling, dipole antenna array, human head imaging, loop + dipole antenna, magnetic resonance imaging, multi-channel arrays, ultra-high field

## Abstract

For ultra-high field and frequency (UHF) magnetic resonance imaging (MRI), the associated short wavelengths in biological tissues leads to penetration and homogeneity issues at 10.5 tesla (T) and require antenna transmit arrays for efficiently generated 447 MHz B_1_^+^ fields (defined as the transmit radiofrequency (RF) magnetic field generated by RF coils). Previously, we evaluated a 16-channel combined loop + dipole antenna (LD) 10.5 T head array. While the LD array configuration did not achieve the desired B_1_^+^ efficiency, it showed an improvement of the specific absorption rate (SAR) efficiency compared to the separate 8-channel loop and separate 8-channel dipole antenna arrays at 10.5 T. Here we compare a 16-channel dipole antenna array with a 16-channel LD array of the same dimensions to evaluate B_1_^+^ efficiency, 10 g SAR, and SAR efficiency. The 16-channel dipole antenna array achieved a 24% increase in B_1_^+^ efficiency in the electromagnetic simulation and MR experiment compared to the LD array, as measured in the central region of a phantom. Based on the simulation results with a human model, we estimate that a 16-channel dipole antenna array for human brain imaging can increase B_1_^+^ efficiency by 15% with similar SAR efficiency compared to a 16-channel LD head array.

## 1. Introduction

In magnetic resonance imaging (MRI), the static magnetic field (B_0_) creates an energy difference between the spins aligned with the magnetic field (parallel) and those aligned against the magnetic field (anti-parallel) [1,2]. The relative fraction of spins in each state follows a Boltzmann distribution, and the greater the static magnetic field strength, the larger the relative number of parallel spins and the stronger the MR signal. As the field strength of MRI systems increases, the associated spin resonance frequency and required radiofrequency (RF) energy for spin excitation increases in biological tissue. The resonance frequency for proton-based MR imaging follows the gyromagnetic ratio of 42.57 MHz⋅T^−1^ [3,4,5,6]. At 7 tesla (T), this results in an MRI operating frequency approaching 300 MHz—which is the lower range of the ultra-high frequency (UHF) band of the electromagnetic (EM) spectrum. This is the reason why the wider MRI community has embraced the term “UHF” to interchangeably describe ultra-high fields (7 T and higher) and ultra-high frequencies. 

The associated increase in MR signal strength results in an increased signal-to-noise ratio (SNR), and is the main driver behind the push for higher static magnetic fields. The wavelength of the EM waves employed for resonant spin excitation of proton signals at UHF (i.e., 447 MHz at 10.5 T) in tissue is shorter compared to the object size. UHF RF excitations cause constructive and destructive interference patterns in the human body and results in significant field inhomogeneities [7,8,9]. Regardless of this limitation, the demand for UHF MRI continues to increase due to its ability to produce images with higher SNR, and increased spatial and temporal resolution compared to common lower field clinical MRI systems, such as 1.5 T and 3 T [10,11,12,13,14].

To address the limitations of UHF MRIs, several dipole and monopole antenna array designs have been explored as RF coils, which are the electrical structures transmit and/or receive RF signals [15,16,17,18,19,20,21,22]. RF coils are evaluated in terms of B_1_^+^ fields (defined as the transmit RF magnetic (B) field generated by the RF coils) and specific absorption rate (SAR) which is defined as the RF power absorbed per unit mass (W/kg). For patient safety purposes, 3D full-wave EM simulation with non-uniform tissue models are performed to calculate the electric (E) fields and SAR associated with the RF transmitter [23,24]. For UHF MRI, RF safety and SAR calculations are the most important factors to consider how SAR is used to estimate how much temperature change (∆T) occurs in specific regions of the sample tissue. The International Commission on Non-Ionizing Radiation Protection (ICNIRP) has shown that over a wide range of frequencies and for both near and far field exposure, 10 g SAR (which means SAR values over any 10 g) is better correlated with ∆T than that of 1 g SAR (which means SAR values over any 1 g), and thus we evaluate 10 g SAR [25]. 

In a previous publication [26], we combined loop and dipole antenna elements to create a 16-channel loop + dipole (LD) antenna array for 447 MHz human head imaging applications. This array was configured with eight LD for a combined total of sixteen channels and was compared with an 8-channel loop-only array and an 8-channel dipole-only antenna array to compare B_1_^+^ field efficiency (defined as B_1_^+^ field normalized by the square root of net input power), SNR, and SAR efficiency (defined as B_1_^+^ field normalized by the square root of the maximum 10 g SAR occurring anywhere in the object). The 16-channel LD array showed improvements in SAR efficiency compared to the 8-channel arrays. Previously, the LD array concept was also successfully developed for 7 T body applications, and achieved the desired deep signal penetration depth [27,28]. It was concluded that dipoles are well suited for UHF applications due to greater depth and the symmetric B_1_^+^ field patterns. At 10.5 T, tighter spacing of dipoles without decoupling circuitry is possible, and high channel count (≥8) dipole antenna arrays are being pursued—particularly for UHF body applications [29,30].

In this article, we evaluate a 16-channel dipole antenna-only array for human head applications. We compared 16-channel dipole and loop + dipole antenna arrays with a phantom in simulation and experiment. Then, we simulated the arrays with a human head model for whole brain imaging evaluation. These phantom results allow direct evaluation of how closely the simulated and experimental results can be matched. We then simulated both arrays with a human head model. Simulations with human head models were used to help validate the SAR levels before an in-vivo experiment. For the evaluation of each array, we initially compared the coupling and B_1_^+^ efficiency between an LD set and two-channels of dipole antennas. Coupling between dipole antennas is the major hurdle for a human head sized dipole array which has 16 or more channels. Scattering (S)-parameters were measured to compare coupling. For 8-channel head arrays, element decoupling values could be improved with a variety of innovative decoupling circuitry options [31,32,33,34]. However, for the dense head arrays, such as the 16-channel arrays presented here, the additional circuitry is very challenging or impractical to incorporate in practice.

To evaluate correlation among channels, the S-parameters of the arrays were measured with a 16-channel network analyzer, and noise covariance maps were experimentally acquired utilizing the 10.5 T MRI scanner. We calculated 10 g SAR and SAR efficiency for all arrays from the EM simulation, which were utilized for the safety validation. Finally, we evaluated the simulated B_1_^+^ efficiency, 10 g SAR, and SAR efficiency of the arrays with the human head model (“Duke”, 34 year old male) from the Virtual Family [35] for human head applications.

## 2. Materials and Methods

### 2.1. Comparison of Coupling and B_1_^+^ Efficiency between an LD Set and Two Dipole Antennas

**Figure 1:**
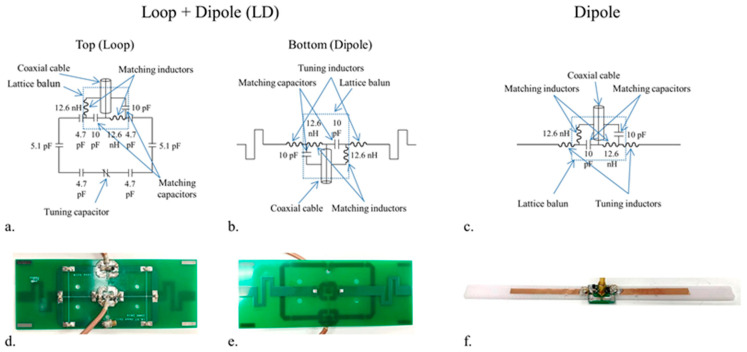
Schema (**a**–**c**) and photographs (**d**–**f**) of an loop + dipole (LD) set and a dipole antenna. A single set of an LD antenna was printed on a two-side FR4 board (top view: a loop (**d**), and bottom view: a dipole (**e**)). The matching circuits of the LD and the dipole antenna were configured with a lattice balun matching circuit to reduce the sheath current.

A lattice balun matching circuit was used to reduce the sheath currents for each LD (Figure 1a,b) set and dipole antenna (Figure 1c). A rectangular loop coil (Figure 1d) which has a size of 6 cm × 10 cm, and a dipole antenna (Figure 1e) which was 18 cm in length, were printed on double sided FR4 printed circuit boards (Advanced circuits, Maple Grove, MN, USA) [26]. For the tuning of each individual loop at 447 MHz, four 4.7 pF, two 5.1 pF ceramic capacitors (100B series, American Technical Ceramics, Huntington Station, NY, USA), and one variable capacitor (JZ200HV, Knowles Voltronics, Cazenovia, NY, USA) were used. The dipole antenna (Figure 1e) of the LD set was shortened to 18 cm by combining dipole fractionation and inductor-shortening at the feed point [36]. For the dipole antenna, as shown in Figure 1f, the length was shortened to 18 cm with just the feed-point inductors.

**Figure 2:**
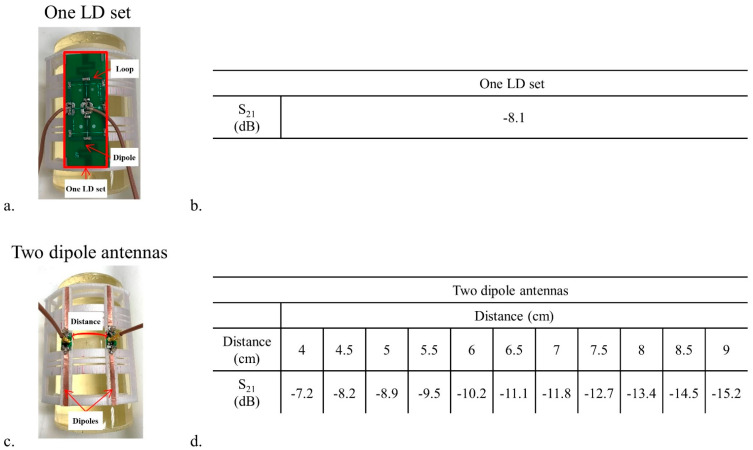
Photographs of an LD set (**a**) and two dipole antennas (**c**) on the phantom. S_21_ values between the loop and the dipole antenna in the LD set (**b**) and two dipole antennas (**d**) were measured through the circumferential center-to-center distance from 4 cm to 9 cm.

**Figure 3:**
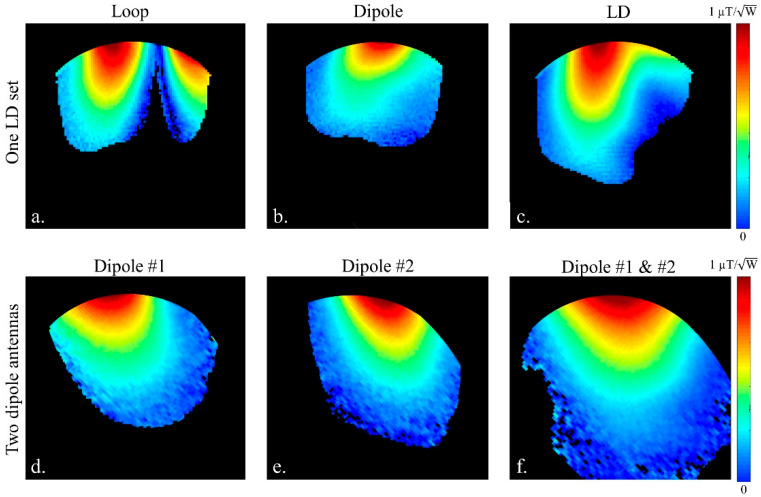
Experimental B_1_^+^ efficiency comparison between an LD set and two dipole antennas with 4 cm circumferential center-to-center distance between the channels. Each individually driven B_1_^+^ efficiency map by a loop (**a**) and a dipole (**b**) of the LD set was acquired. And, in figure (**c**), both two elements of the LD set were driven simultaneously and normalized by the net input power for the comparison of the B_1_^+^ efficiency. For the comparison with the LD set, B_1_^+^ efficiency maps of each dipole antenna (**d**,**e**) of two dipole antennas and two activated dipole antennas (**f**) were also obtained. Each map was acquired at the center slice of the phantom in the transaxial plane.

One LD set and two-channels of the inductor-shortened dipole antenna were chosen for the comparison of coupling and B_1_^+^ efficiency. For coupling measurements, all input reflection (S_11_) and transmission coupling (S_21_) coefficients were measured in dB scaled values using a 16-channel network analyzer (ZNBT8, Rohde & Schwarz, Munich, Germany). S_21_, between the LD set elements (Figure 2a,b), and two dipole antenna elements (Figure 2c,d) spaced 4 cm to 9 cm apart from one another, were measured on a cylindrically shaped phantom. As shown in Figure 2a,c, the LD set and two dipole antennas were positioned with a 5 cm gap between the phantom surface and antenna elements. The cylindrical phantom (diameter = 18 cm and height = 30.5 cm) was filled with a sucrose doped saline solution for validation testing. A dielectric assessment kit (DAKS-12, SPEAG, Zurich, Switzerland) was used to measure the electrical properties of the cylindrical phantom (ε_r_ = 49 and σ = 0.6 S/m) at 447 MHz [37]. For comparison of B_1_^+^ efficiency (Figure 3) between an LD set and two dipole antennas, the circumferential center-to-center distance between two dipole antennas was set to 4 cm, the same gap between adjacent elements in the 16-channel dipole antenna array.

### 2.2. Setup for the Experiments

All experimental data were obtained with a 10.5 T whole body magnet (Agilent, Santa Clara, CA, USA), operated by a Siemens 10.5 T console configured with 16-channel independent parallel transmitters (pTx) and 32-channel receivers (Siemens Healthineers, Erlangen, Germany). The pTx system was connected to each array using a custom 16-channel transmit/receive interface box. To calculate B_1_^+^ efficiency, the flip angle for a given pulse must be calculated. An actual flip angle imaging (AFI) sequence [38] used the signal levels from two interleaved images with different TRs to calculate the flip angle for each pixel. The AFI sequence (TR_1_/TR_2_ = 25/115 ms, TE = 3.39 ms, nominal flip angle = 60°, GRAPPA (R = 2), and resolution = 2 mm × 4 mm × 6 mm) was utilized to calculate the B_1_^+^ field maps within the phantom. The flip angle (α) with short TR_1_ and TR_2_ was calculated by [38]:

(1) $$\alpha =\mathrm{arcos}\frac{\mathrm{rn}-1}{n-r}\;$$

where α = flip angle and n = TR_2_/TR_1_. The signal from TR_1_ (S_1_) and the signal from TR_2_ (S_2_) are proportional to:

(2) $$S_{1,2}=M_{z1,2}\;\exp\;(-\mathrm{TE}/T_{2}*)\;\sin\;\alpha$$

where M_z_ is the z-directional magnetization. The signal ratio can be expressed as:

(3) $$r=S_{2}/S_{1}=\frac{1-E_{1}+\left(1-E_{2}\right)\;E_{1\;}\cos\;\alpha }{1-E_{2}+\left(1-E_{1}\right)\;E_{2\;}\cos\;\alpha }$$

where E_1,2_ = exp (−TR_1,2/_T_1_). B_y_ applying the first-order approximation to exponential terms, equation [3] can be simplified for short TR_1_ and TR_2_:

(4) $$r\;\approx \frac{1+n\;\cos\alpha }{n+\cos\alpha }\;$$

The flip angle was converted to B_1_^+^ with $\alpha =2{\pi \gamma B}_{1}^{+}\tau \;,$ where τ is the width in seconds of the RF pulse [39]. Noise covariance matrices of the 16-channel LD and dipole antenna arrays were also acquired experimentally [40].

### 2.3. Construction of the 16-Channel LD and Dipole Antenna Arrays

**Figure 4:**
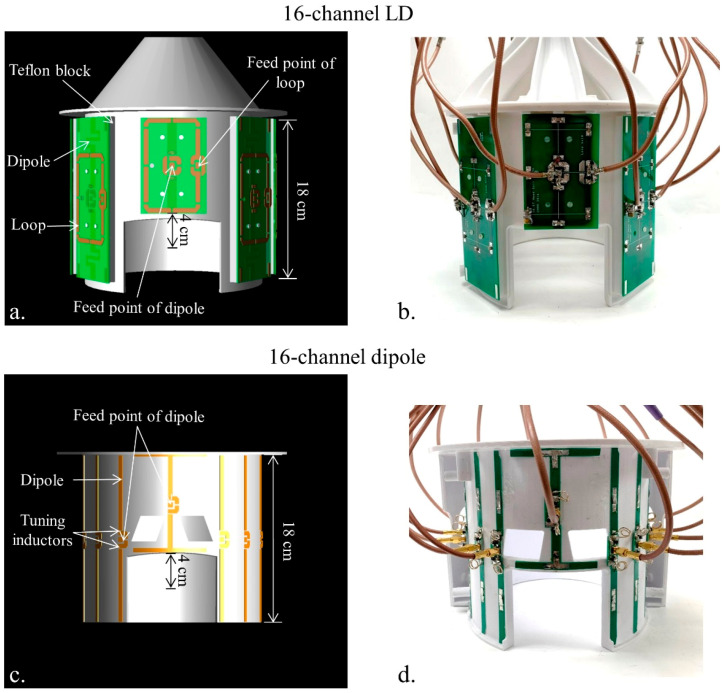
3D modeling (**a**,**c**) and photographs (**b**,**d**) of the built 16-channel LD and dipole antenna arrays. The anterior element of the LD array and dipole antenna array were shifted 4 cm superior.

As shown in Figure 4, elliptically shaped formers with a minor axis of 200 mm and a major axis of 220 mm were modeled and printed by a 3D printer (F410, Fusion3 Design, Greensboro, NC, USA). The 16-channel LD array has eight equally spaced channels with an 8.8 cm circumferential center-to-center distance between sets. The eight loops and eight dipole antennas of the 16-channel LD array were mounted to Teflon blocks which were fixed to the printed housing frame (thickness: 0.4 cm) [26]. Since the dimension of the array former is same for both antenna architectures, the 16-channel dipole antenna array has a 4 cm gap between each of the sixteen individual channels and does not contain any decoupling circuitry.

At the anterior position of the array, one set (one pair of a loop and a dipole antenna) of the 16-channel LD array (Figure 4a,b) and one channel of the 16-channel dipole antenna array (Figure 4c,d) were located 4 cm superior compared to the other channels and sets, respectively. The anterior dipole of the LD array was shortened to a total length of 10 cm with tuning inductors positioned at the feed point. However, a tangential-shaped antenna was selected with a pair of tuning inductors to increase the electrical length of the anterior channel of the dipole antenna array. All values of S_11_ and the S_21_ values of the first nearest and the second nearest channels were summarized in Table 1.

### 2.4. Setup for the Simulation and Numerical Analysis

We performed finite-difference time-domain (FDTD) simulations of these transmitters using XFdtd (REMCOM, State College, PA, USA) to obtain calculated steady-state B- and E-field distributions. Simulations were performed with a cylindrical phantom, as well as a 3D tissue voxel head and shoulder model. The 10 g SAR map for each array was calculated using:

(5) $$\mathrm{SAR}=\frac{\sigma_{x}}{2\rho_{x}}E_{x}^{2}+\frac{\sigma_{y}}{2\rho_{y}}E_{y}^{2}+\frac{\sigma_{z}}{2\rho_{z}}E_{z}^{2}$$

where σ is the electrical conductivity (siemens/m), ρ is the material density (kg/m^3^), and E_x,y,z_ are the vector components of the E-field for each voxel of the simulation, for both the phantom and the human model. Simulated B_1_^+^ efficiency was calculated by:

(6) $${B_{1}}^{+}=\left|\frac{B_{x}+{\mathrm{iB}}_{y}}{2}\right|$$

where B_x_ and B_y_ are the complex amplitudes of x- and y-oriented RF magnetic fields, respectively [38]. Then, B_1_^+^ efficiency (obtained by the simulation and the experiment) and SAR efficiency were calculated by the net input power and peak 10 g SAR, respectively. Post-processing was performed with custom Matlab (The Mathworks, Inc., Natick, MA, USA) code to compare the performance between the two arrays. For a quantitative comparison, one region of interest (ROI) in each transaxial plane of each map was chosen to compare the highest B_1_^+^ efficiency, 10 g SAR, and SAR efficiency in the phantom and the human model. The values from each ROI were shown below each coronal figure and summarized in Table 2.

## 3. Results

### 3.1. Comparison of Coupling and B_1_^+^ Efficiency with an LD Set and Two-Channel Dipole Antennas

Similar transmission coefficients (S_21_) were observed between an LD set (Figure 2b) and the two-channel dipole antennas with the 4.5 cm distance (Figure 2d). As expected, the S_21_ between two dipole antennas in Figure 2d decreased as the distance between the dipole antennas increased, demonstrating the reduced coupling with larger spacing. The S_21_ of two dipole antennas with 4 cm distance showed high correlation (−7.2 dB); however, this is an acceptable value if the antennas perform well enough in terms of the B_1_^+^ efficiency.

Each subplot of Figure 3 was obtained at the center transaxial slice of each antenna. The loop (Figure 3a) of the LD set outperformed the dipole antennas (Figure 3b,d,e) at the peripheral area of the phantom. However, the dipole antennas in Figure 3c,d showed deeper penetration compared to the loop (Figure 3a) and the dipole antenna (Figure 3b) of the LD set. The LD set (Figure 3c) showed higher B_1_^+^ efficiency at the peripheral area of the subject, though lower penetration depth compared to the two-channel dipole antennas (Figure 3f).

### 3.2. S-parameters and Noise Covariance of the 16-Channel LD and Dipole Antenna Arrays

**Figure 5:**
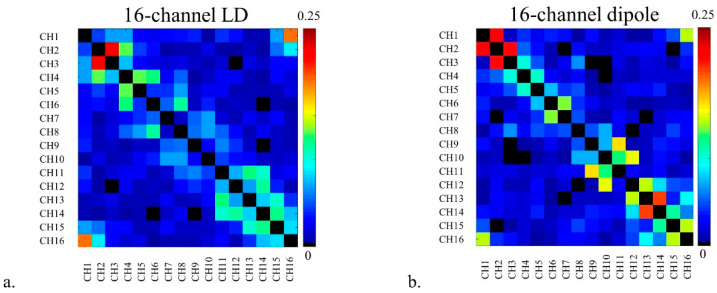
Noise covariance matrices of the 16-channel LD (**a**) and dipole antenna (**b**) arrays. The distance between channels was 8.8 cm for the LD array and 4 cm for the dipole antenna array, respectively.

Table 1 indicates a summary of S-parameters with two arrays including S_11_, S_21_, and S_31_ values when two arrays were loaded with the phantom. S_21_ and S_31_ were in the range from −8.1 dB to −24.2 dB and from −12.4 dB to −22.4 dB for the 16-channel LD array, and from −7.1 dB to −18.3 dB and from −13.8 dB to −26.9 dB for the 16-channel dipole antenna array, respectively. As shown in Figure 5, noise covariance matrices between the 16-channel LD and dipole antenna arrays were obtained in an MR experiment. The noise covariance for the 16-channel LD array (max: 0.22) in Figure 5a also showed the preferred overall lower values compared to the 16-channel dipole antenna array (max: 0.22) in Figure 5b. Noise covariance of each array was achieved with an acceptable value, which is lower than 0.25.

### 3.3. Comparison of B_1_^+^ Efficiency, 10 g SAR, and SAR Efficiency with the 16-Channel Arrays

**Figure 6:**
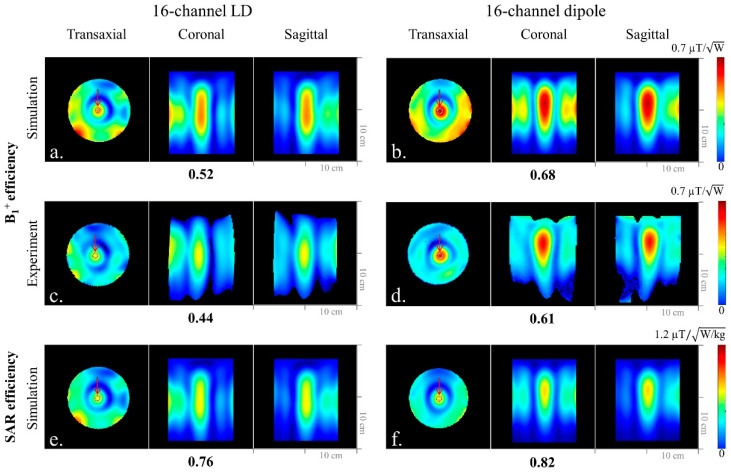
Simulated B_1_^+^ efficiency (**a**,**b**), experimental B_1_^+^ efficiency (**c**,**d**), and SAR efficiency (**e**,**f**) maps of the 16-channel LD and dipole antenna arrays with a cylindrical phantom in the transaxial, coronal, and sagittal planes, respectively. The number below each subfigure was the value from the ROI indicated in each transaxial plane.

Simulated (Figure 6a,b) and experimental (Figure 6c,d) B_1_^+^ efficiency maps of each array are shown. In both the EM simulation and the experiment, the dipole antenna array showed 24% higher B_1_^+^ efficiency over the LD array. In terms of SAR efficiency, the simulation showed 7% improvement for the dipole antenna array (Figure 6f) compared to the LD array (Figure 6e).

**Figure 7:**
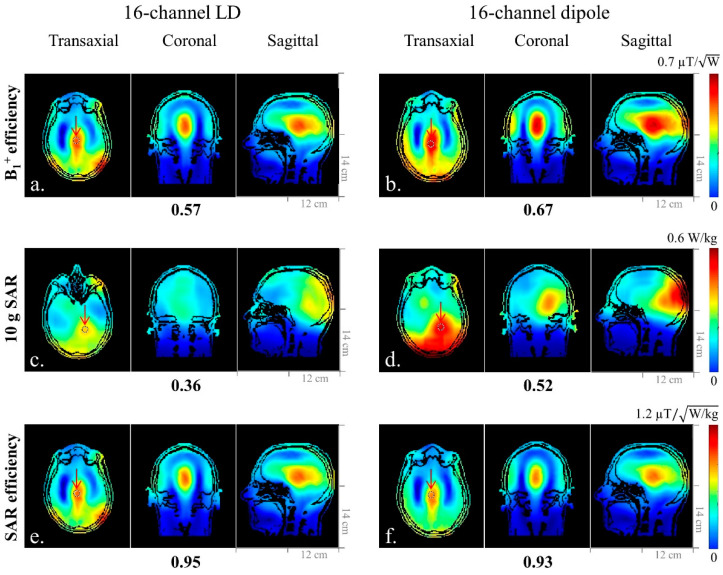
Simulated B_1_^+^ efficiency (**a**,**b**), 10 g SAR (**c**,**d**), and SAR efficiency (**e**,**f**) maps of the 16-channel LD and dipole antenna arrays with a human head model in the transaxial, coronal, and sagittal planes. The number below each subfigure was the value from the ROI indicated in each transaxial plane. The 16-channel LD and dipole antenna arrays showed similar SAR efficiency values.

In a human model, B_1_^+^ efficiency of the dipole antenna array (Figure 7b) showed 15% higher improvement compared to the LD array (Figure 7a). Peak 10 g SAR values measured on the human model were 0.36 W/kg and 0.52 W/kg for the LD (Figure 7c) and dipole antenna (Figure 7d) arrays, respectively. The dipole antenna array showed a 31% higher peak 10 g SAR value compared to the LD array, which resulted in slightly lower SAR efficiency values with the dipole antenna array (0.93) compared to the LD array (0.95) in Figure 7f,e, respectively.

## 4. Discussion

The comparison between a 16-channel combined array of eight loops and eight dipoles and a 16-channel dipole-only antenna array has been evaluated at 10.5 T with a phantom and a human head model. The dipole antenna array showed higher coupling between adjacent channels due to the physically closer distance among the channels compared to the LD array. However, the inter-channel coupling for a dipole antenna array at a higher operating magnetic field could be less of a prohibitive factor compared to lower magnetic field MRI RF coils, due to an increased coupling to the load and narrower RF coil apparatus profiles. Hence, the 16-channel dipole antenna array showed ~24% higher B_1_^+^ efficiency with the phantom in the ROI compared to the 16-channel LD array in both the simulation and the experiment. Consistently, the B_1_^+^ efficiency with the human model showed a 15% improvement with the dipole antenna array compared to the LD array.

From the previous publication [26], the 16-channel LD array with the PVP-agar gel mixture (ε_r_ of 49 and σ of 0.69 S/m) phantom showed the lower peak 10 g SAR and improved SAR efficiency compared to the 8-channel dipole antenna only and the 8-channel loop-only arrays. In this article, we expanded the comparison with the 16-channel LD and dipole antenna arrays. The 16-channel dipole antenna array with the sucrose water phantom (ε_r_ of 49 and σ of 0.6 S/m) showed improvement in SAR efficiency compared to the 16-channel LD array. However, with a human model, the 16-channel dipole antenna array did not show higher SAR efficiency compared to the 16-channel LD array. The distance between antennas of both arrays and the human model is closer compared to the phantom, especially in the anterior to posterior direction. Substantially increased peak 10 g SAR was observed due to the closer distance between dipole antennas and the human model compared to the distance between dipole antennas and the phantom. This results in similar SAR efficiency between the LD and dipole antenna arrays.

The spacing among the coaxial cables gets more limited as the number of channels increases. To reduce the interaction between coaxial feed cables and dipole antennas for the 16-channel array, we equipped each channel with a lattice balun matching circuit. For the 16-channel dipole antenna array, we observed that the coaxial cables and the dipole antennas interacted even with the inclusion of the balun circuits. Therefore, we provided the maximal distance between the coaxial cables and antennas. However, this is not a practical solution for the coaxial feed cable routing in most clinical applications.

All of the demonstrations in this manuscript are designed to optimize RF coils for human head imaging. However, many valuable clinical applications such as diffusion tensor imaging (DTI) [41,42] and functional MRI (fMRI) [43,44] achieved with echo planar imaging (EPI) [45,46] sequences can be sensitive to eddy currents generated by RF coils and RF hardware. We did not include any eddy current test results in this manuscript; however, we did evaluate the 16-channel dipole and loop + dipole antenna arrays with EPI sequences within the 10.5 T MRI scanner and we did not observe any eddy current artifacts.

## 5. Conclusions

Here we compared two different types of 16-channel arrays: combined loop + dipole and dipole only antennas. With similar dimensions, the 16-channel dipole antenna array showed some improvement of B_1_^+^ efficiency compared to the 16-channel LD array. Detailed studies demonstrating advantages of dipoles for MR applications at UHF were shown by various researchers [29,47]. Raaijmakers et al. [18], had demonstrated that the favorable Poynting vector of radiative type antennas support deeper penetration into the sample compared to other antenna types, such as arrays of loops [48,49] or microstrip elements [50,51]. For a dipole head array at 10.5 T, our data indicates that radiation pattern and the Poynting vector of dipoles is preferable for deeper structures and supports improved B_1_^+^ phase shimming. Additionally, the simplicity of construction is another distinct advantage of dipole antenna arrays.

SAR efficiency results differ depending on the distance between the subject and the arrays [47]. While it is difficult to allow subject specific geometrical head antenna arrangements within the typical MR safety framework (SAR evaluations rely on a known coil geometry), using the former of a geometrically adjustable 7 T stripline array, which our group had previously reported [50], represents the possibility to construct a head coil former with adjustable radial distance dipole elements [52].

In the future, we plan to design a dual row 32-channel LD array consisting of shortened dipoles and loops. Additionally, we will compare this array with the presented 16-channel dipole antenna array to evaluate possible advantages regarding B_1_^+^ efficiency and B_1_^+^ field control. We also plan to further study efficient and safe coaxial feed cable routing for clinical research, and will evaluate array combinations of loop, dipole antennas, and sleeve antennas [20].

## Figures and Tables

**Table 1:** Measured S-parameters of the 16-channel LD and dipole antenna arrays. S_11_ is the reflection coefficient of each channel, and the S_21_ and S_31_ are the coupling coefficients between adjacent elements and elements separated by an element between them (second nearest), respectively.

	16-Channel LD	16-Channel Dipole
S_11_ (Reflection)	−12.2 dB to −24.3 dB	−14.2 dB to −29.8 dB
S_21_ (Coupling) -Among the adjacent	−8.1 dB to −24.2 dB	−7.1 dB to −18.3 dB
S_31_ (Coupling) -Among second nearest	−12.4 dB to −22.4 dB	−13.8 dB to −26.9 dB

**Table 2:** Summary of B_1_^+^ efficiency, peak 10 g specific absorption rate (SAR), and SAR efficiency between the 16-channel dipole and LD arrays with a phantom and a human model in the region of interest (ROI) positioned on the highest value. The experimental data were obtained only for the B_1_^+^ efficiency in the phantom.

		16-Channel LD	16-Channel Dipole
Phantom	B_1_^+^ efficiency (μT/√W) -Simulation	0.52	0.68
	B_1_^+^ efficiency (μT/√W) -Experiment	0.44	0.61
	Peak 10g SAR (W/kg)	0.47	0.65
	SAR efficiency (µT$/\sqrt{W/\mathrm{kg}}$)	0.76	0.82
Virtual human head model (Duke)	B_1_^+^ efficiency (μT/√W)	0.57	0.67
	Peak 10g SAR (W/kg)	0.36	0.52
	SAR efficiency (µT$/\sqrt{W/\mathrm{kg}}$)	0.95	0.93

## Data Availability

The data presented in this study are available on request from the corresponding author.
